# The promise of a “cure” for HIV: implications for the future of PEPFAR‐supported HIV programmes

**DOI:** 10.1002/jia2.26206

**Published:** 2024-01-09

**Authors:** Elliot Raizes, John Blandford, Joseph M. McCune, Mark Dybul

**Affiliations:** ^1^ United States Centers for Disease Control and Prevention Atlanta Georgia USA; ^2^ Bill and Melinda Gates Foundation Seattle Washington USA; ^3^ Georgetown University Medical Center Washington DC USA

1

As we commemorate the 20th anniversary of the U.S. President's Emergency for AIDS Relief (PEPFAR), there is much to celebrate: over 20 million persons with HIV are receiving life‐saving antiretroviral treatment (ART) in over 50 PEPFAR‐supported countries [1]. However, external financial support for HIV in low‐ and middle‐income countries (LMICs) remains flat, and overall resources are estimated to be at least $8 billion short of the $29 billion needed to reach the 2025 goals of 95‐95‐95^1^ [2].

Even if these goals are realized, they will only be sustained if resources are provided indefinitely. The prospect of HIV “cure,” that is durable ART‐free suppression of HIV, represents an attractive alternative to such ongoing resource commitments—beckoning investment in an intervention that would extend lives, reduce new HIV acquisitions and obviate the need for continued ART.

With this in mind, we read with interest the report in this journal by Guzauskas and Hallet [3] on “the long‐term impact and value of curative therapy for HIV.” Modelling the health benefits of such an intervention, they found that “HIV cure” would more rapidly lower HIV prevalence, prevent future acquisitions over time, increase life years in the population and reduce AIDS‐related deaths when compared to ART alone even in LMICs. These benefits were even more pronounced in settings with higher HIV burdens and lower ART adherence. They also found that such an intervention would be cost‐effective for the duration of HIV patients’ lifetimes if price aligned with a country's per capita income and disease burden.

As has been pointed out [4, 5], even though the timeline to successive generations of curative interventions remains indeterminate, there are important considerations to be addressed sooner rather than later. For instance, the development of a target product profile that envisions application to populations in diverse settings, including LMICs, is an important step requiring updates informed by scientific advances [5]. Though efforts to engage key stakeholders and build capacity enabling widespread access to these interventions in LMICs are already underway, they must in the future expand as candidate cures move to the clinic [5, 6]. Of note, many of the activities now supported by PEPFAR for ART scale‐up and treatment monitoring are perfectly suited to the introduction of an HIV cure intervention.

We applaud the work of Guzauskas and Hallet for advocating for early anticipation of resource needs for cure interventions to ensure widespread access in LMICs. For the investment to be cost‐effective and adopted across countries with diverse income levels and HIV disease burdens, tailored pricing might be offered that accounts for the current costs of delivering lifelong ART, the value of improved health among those living with HIV and projected savings from averted HIV acquisitions. New approaches to financing the implementation of an HIV cure will be needed so that implementation can be global, equitable and appropriately paced. PEPFAR, along with other donors and stakeholders, would seem to be well poised to further catalyse this and other efforts, working with country governments, regulatory agencies and the community to realize the promise of a cure for HIV, thereby accelerating the elimination of this deadly virus around the world.

## COMPETING INTERESTS

The authors have no competing interests to declare.

## AUTHORS’ CONTRIBUTIONS

ER: Original draft of the manuscript and subsequent edits. JB: Contributed to the economic issues raised. JMMc: Contributed to the overall message of the manuscript and the specific technical components of HIV cure. MD: Contributed to the overall message of the manuscript and the specific technical components of HIV cure with a focus on LMICs.

## DISCLAIMER

The findings and conclusions in this manuscript are those of the authors and do not necessarily represent the official position of the United States Centers for Disease Control and Prevention.

## Data Availability

Data derived from public domain resources.

## ENDNOTES

1 95% of PWH know their HIV status, 95% of PWH who know their status are on ART, 95% of PWH on ART are virally suppressed.
